# Empowering through structured boundaries: an integral model for fostering balanced eating and nutritional well-being

**DOI:** 10.1007/s44162-025-00145-3

**Published:** 2026-01-13

**Authors:** Citrine Elatrash, Colleen Macke, Jenna Shi, Theresa Wilson, Sarah H. Elsea, Stephanie Sisley

**Affiliations:** 1 Department of Pediatrics, USDA/ARS Children’s Nutrition Research Center, Baylor College of Medicine, 1100 Bates St., Houston, TX 77030 USA; 2 Department of Pediatrics, Division of Endocrinology, Baylor College of Medicine, Houston, TX USA; 3 Rice University, Houston, TX USA; 4 Department of Molecular and Human Genetics, Baylor College of Medicine, Houston, TX USA

**Keywords:** Smith-Magenis syndrome, Food-related behaviors, Obesity, Behavior management

## Abstract

**Purpose:**

Treating obesity in complex disorders requires individualized health management plans. Effective obesity management in the Smith-Magenis syndrome (SMS) population has multiple inherent barriers related to their cognitive impairments, anxiety, behavioral disturbances, and sleep disorders. However, to date, no research exists related to strategies caregivers successfully employ to help individuals with SMS manage their food intake and mitigate associated behaviors.

**Methods:**

We performed a qualitative thematic analysis of group interviews from 23 caregivers representing 21 individuals with SMS. Hybrid thematic analysis revealed that successful strategies employed by caregivers in this unique population revolved around a global theme of *Empowering Through Structured Boundaries*.

**Results:**

Four themes emerged: Fostering Balanced Eating, Structuring Environments, Setting the Stage for Success, and Managing Challenges and Resistance. Together, our analysis shows that successful strategies utilized by caregivers often involve setting up explicit boundaries, both literal and figurative, that provide choice empowerment to the individual with SMS. Additionally, anticipatory guidance, deflection, and positive language were utilized throughout different contexts.

**Conclusions:**

These data are the first to define and disseminate a variety of successful strategies utilized by caregivers of individuals with SMS to decrease food-related disruptive behaviors.

**Supplementary Information:**

The online version contains supplementary material available at 10.1007/s44162-025-00145-3.

## Introduction

Treating obesity in complex disorders requires individualized health management plans. Smith-Magenis syndrome (SMS) is a genetic condition that results from loss of chromosome 17p11.2 or a pathogenic variant in *RAI1* [15, 16]. In addition to obesity, individuals with SMS have a variety of other concerns, including cognitive impairments, anxiety, behavioral disturbances, and sleep disorders. Interestingly, unlike other genetic forms of obesity, individuals with SMS often develop obesity in their middle childhood years [16].

We have previously published that food is a significant and daily focus for many individuals with SMS [7]. Our previous work in this area focused on the behaviors observed in individuals with SMS and revealed that they have impaired satiety (measured through the Food-related Behaviors Questionnaire) equivalent to individuals with Prader-Willi syndrome (PWS) [1] and that maladaptive food-related behaviors are elevated in individuals with obesity and those taking certain types of medications [8]. Additionally, we have published that individuals with SMS perseverate on food but interestingly, have unique food-related behaviors that require significant gatekeeping by their caregivers (for example only overeating bananas or condiments) [7]. However, the strategies caregivers used to help prevent, mitigate, or react to food-related behaviors were not well understood.

Given the unique food-related behaviors observed in the SMS population, along with the underlying circadian, behavioral, and cognitive struggles associated with the syndrome, treating obesity in the SMS population presents multiple inherent barriers. Currently, overcoming these barriers occurs at an individual level, with caregivers and medical providers using word-of-mouth to disseminate effective strategies, as there has been no targeted study of effective strategies in this population. Identifying and understanding beneficial strategies in this rare population is crucial to guiding both caregivers and healthcare providers in successfully treating obesity in individuals with SMS. While our previous work focused on the behaviors evident in individuals with SMS, in this study, we used a qualitative approach to focus on how caregivers respond to manage or mitigate these behaviors.

## Materials and methods

This study was approved by the Baylor College of Medicine Institutional Review Board and was performed in accordance with the ethical standards of the 1964 Declaration of Helsinki and its later amendments. We performed qualitative analyses of caregiver strategies abstracted from interviews collected for our previous paper, determining the expression and impact of food-related behaviors in the SMS population [7]. The key components of the methodological approach are outlined below. Clinical Trial Number: not applicable.

### Study design and participants

A qualitative study design was employed using semi-structured interviews and focus groups [7, 11, 18]. Caregivers (*N =* 23) representing 21 individuals with SMS were recruited from the Parents and Researchers Interested in SMS (PRISMS) 2022 national meeting and social media platforms. Participants gave verbal consent prior to participation.

### Interview process

An interview script was developed based on the Socio-Ecological Model and previous work in SMS, focusing on the food-related behaviors in individuals with SMS and the strategies their caregivers used to mitigate them. Our script has been previously published [7]. Utilizing open-ended questions, we explored the following factors:

- How food influences behavior currently or in the past
- Strategies employed to limit food intake currently or in the past
- Situational factors affecting food-related behaviors currently or in the past
- Specific techniques used during mealtimes (e.g., portioning, environmental controls, and communication strategies) currently or in the past

Interviews were conducted via focus groups of caregivers of individuals with SMS in four different groups: two groups in person at the PRISMS 2022 International Conference and two groups through a video conference software. Two individuals from our study team who were trained in qualitative methods conducted the interviews. Each interview was digitally recorded, transcribed verbatim, and anonymized to ensure confidentiality [2, 7]. Each transcript was cross-referenced with video recordings to assign participant identifiers and verify accuracy.

### Data analysis

Qualitative data were analyzed using a hybrid thematic analysis approach [3], integrating both deductive and inductive coding methods. Three trained coders reviewed the transcripts repeatedly to gain a comprehensive understanding of participant responses. An initial code framework was developed deductively from the interview topics and preliminary insights. Each transcript was then coded independently by three coders, with new inductive codes added to capture emerging concepts. A detailed codebook documenting coding definitions, decisions, and changes was maintained to ensure analytic transparency and provide an audit trail. The research team met regularly to reconcile discrepancies and reach consensus through inter-coder agreement. While our initial analysis focused on food-related behaviors, for this project, we re-analyzed all mentions of specific strategies from a caregiver strategy lens. During the analysis, emergent subthemes specifically relating to caregiver strategies were added to capture unique practices and adaptive techniques. The frequency and diversity of these strategies were then mapped to form an abstract framework that reflected our integrated model.

### Ethical considerations

The study was approved by the Institutional Review Board of Baylor College of Medicine. All participants were caregivers of an individual with SMS, whose genetic diagnosis of SMS was previously confirmed. All caregivers provided informed consent prior to participation, and efforts were made to ensure that participants’ identities and sensitive data remained confidential.

## Results

A total of 24 caregivers were initially included in the parent study. Of these, 23 caregivers provided responses regarding the strategies they use to manage food-related behavioral issues in individuals with SMS. Participant-defined demographic information for these 23 caregivers and the 21 individuals they represented is summarized in Table 1. The majority of caregivers were female (82.6%). The average age of the caregiver was 49.9 ± 11 years, and the majority identified as non-Hispanic White (74%), followed by Hispanic White (8.7%). Regarding the 21 individuals with SMS, 66.7% were female, with an average age of 19.7 ± 9.9 years. Similar to their caregivers, the majority of the individuals with SMS were non-Hispanic White (85.7%), followed by Hispanic White (9.5%). Most individuals with SMS lived at home (76.2%) with one or both parents, while 14.3% resided in a group home. The remaining 9.5% lived with other relatives or in a host home.

**Table 1:** Demographics of caregivers and individuals with SMS

Demographics of Caregivers of Individuals with SMS	*N =* 23
Female, %	82.6
Age, y	49.9 ± 11.0
Mother	47.9 ± 9.8
Father	62.3 ± 5.7
Other	44.3 ± 16.17
Race/ethnicity, %	
Non-Hispanic White	74
Hispanic White	8.7
Non-Hispanic Black or Hispanic Black	0
Other/no answer	4.3
Relationship to individual with SMS, %	
Mother	69.6
Father	17.4
Other	13.0
Demographics of individuals with SMS at the time of study	*N =* 21
Female, %	66.7
Age, y	19.7 ± 9.9
Age of those living at home with one or both parents	17.5 ± 8.1
Age of those living in a group home	32.3 ± 7.5
Age of those living with other relatives or host home	26.7 ± 12.7
Race/ethnicity%	
Non-Hispanic White	85.7
Hispanic White	9.5
Non-Hispanic Black or Hispanic Black	0
Other/no answer	4.8
Living arrangement %	
At home with one or both parents	76.2
Group home	14.3
Other relatives or host home	9.5

*SMS* Smith-Magenis syndrome

**Fig. 1:**
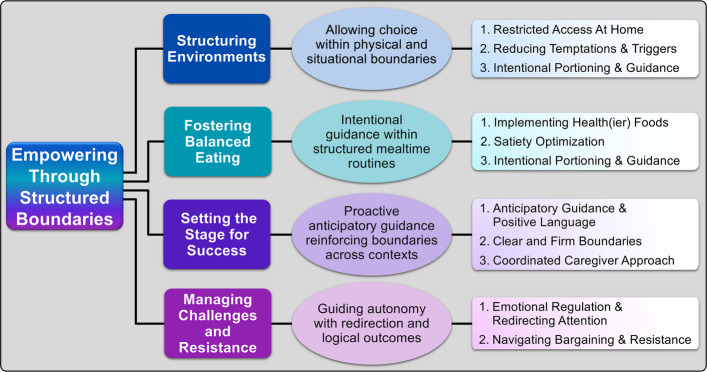
Thematic analysis of strategies employed by caregivers of individuals with Smith-Magenis syndrome to manage food-related behaviors by empowering individuals through reinforcing structure, predictability and autonomy. Ovals describe how each theme contributes to the overall global theme of “*Empowering through structured boundaries*”. Subthemes are enumerated to the right

After analyzing the qualitative data, one global theme emerged (Empowering Through Structured Boundaries) with four organizing themes (Fig. 1): Structuring Environments, Fostering Balanced Eating, Setting the Stage for Success, and Managing Challenges and Resistance. The themes and subthemes are listed and discussed below, organized by frequency. However, given the utility of disseminating the specific wording and strategies utilized by caregivers, we have created a table listing each specific strategy described in our study (Supplemental Table 1).

### Global theme: empowering through structured boundaries

The strategies caregivers employed revolved around a central theme of empowering the individuals in their care by establishing structured boundaries that balanced autonomy with oversight (Fig. 1). Utilizing this approach, caregivers developed specific strategies to structure environments to foster healthy eating, handle attempts from individuals to bypass restrictions, and setup appropriate expectations. These structured approaches aimed to minimize conflict while mitigating unhealthy behaviors, allowing individuals with SMS to engage with food more adaptively.

#### Organizing theme # 1: structuring environments (20/23 [87.0%] representing 19/21 [90.5%] individuals with SMS)

The majority of caregivers restricted access to food and strategically offered food to manage impulsive eating, limiting high-risk foods, and structuring choices. Many of these strategies focused on reducing temptations and triggers, such as avoiding buffets and other high-calorie food options, as well as physical and financial barriers, such as locked cabinets.

#### Restricted access at home (19/23 [82.6%] caregivers representing 18/21 [85.7%] individuals with SMS)

Many caregivers took deliberate steps to manage food access, creating structured environments to prevent overconsumption and impulsive eating at home. Caregivers used restrictive strategies, including gates to prevent access to parts of the home, locking pantries and refrigerators, using keypad-locked snack closets, and designating specific shelves or snack boxes with approved options. The necessity of securing certain desirable food items was described by Caregiver 18: “*If we want something to last more than a day, we have to hide it*.” To achieve total lockdown, Caregiver 8 “*had a refrigerator in the pantry, and the pantry and the refrigerator were locked*.” However, importantly, the restricted access needs changed over time as the same caregiver noted that “*now everything’s open*” because their daughter was able to comply with newer rules around food as she matured. Given the circadian disruption often observed in individuals with SMS, nighttime restriction was important as Caregiver 6 noted “*he’s locked in at night.”* Other caregivers hid unhealthy foods in unexpected places, from ovens to toolboxes. Restricting access was hard for some caregivers initially, but as Caregiver 24 noted, guidance from an SMS webinar shifted their perspective: “*We felt guilty about locking things up, but then we learned it’s in the genetic code. We realized we were doing harm by not putting barriers in place.*”

#### Reducing temptations & triggers (8/23 [34.8%] caregivers representing 8/21 [38.1%] individuals with SMS)

Caregivers found that minimizing exposure to certain tempting foods and triggering eating environments helped reduce impulsive behaviors. Caregiver 4 shared, “*If she doesn’t see it, she doesn’t think about it*.” Some caregivers strategically place healthy alternatives in accessible spots, such as leaving out bananas for nighttime cravings, as Caregiver 21 shared, “*When she gets up at night, I leave a banana out for her. She knows it’s there and will take it instead of searching for other food.*” Although involving individuals with SMS in food planning was successful for many families (noted more below in Theme #2), for individuals with food-related anxiety, the opposite may be true. Caregiver 11 explained, “*What works best is taking him out of the equation completely – no meal prep, no grocery trips. It took years of trial and error, but it helps lessen his stress.*” Additionally, structured seating arrangements were sometimes necessary: Caregiver 22 found, “*My son sits a seat away from the other kids at school because he’ll just grab their food, and it turns into a huge issue*.” Avoiding all-you-can-eat settings was also a common tactic to reduce the triggers of overeating. These strategies illustrate how reducing food-related triggers can create a more manageable eating environment.

#### Artificial financial barriers (3/23 [13.0%] caregivers representing 3/21 [14.3%] individuals with SMS)

To maintain structure and prevent excessive food consumption, some caregivers utilized strategic financial barriers. Caregiver 11 described, “*I’ve been trying to pay with cash everywhere [at the hotel] because the last thing I need is for him to learn he can just charge [food] to the room.*” Similarly, Caregiver 20 shared, “*So we have to limit her money even to access that kind of stuff.*” By enforcing these boundaries, several caregivers created necessary constraints to help regulate food access and prevent overconsumption.

#### Organizing theme # 2: fostering balanced eating (19/23 [82.6%] caregivers representing 17/21 [81.0%] individuals with SMS)

Caregivers of individuals with SMS emphasized the importance of structured strategies to promote balanced eating habits in individuals with SMS. Portion control, encouraging consistent healthy eating, and meal planning were key approaches used to improve diet quality and quantity. Caregivers highlighted the necessity of proactive guidance to reinforce appropriate food choices while maintaining structured yet flexible approaches to mealtime routines.

#### Implementing health(ier) foods (14/23 [60.9%] caregivers representing 14/21 [66.7%] individuals with SMS)

Encouraging individuals with SMS to make healthier food choices was an essential strategy for many caregivers. Caregivers stopped buying many different unhealthy foods and replaced less healthy options with more nutritious alternatives, such as offering water instead of soda and plant-based protein chips instead of traditional snacks. As Caregiver 23 explained, “*We try to go for baked chips or protein chips, things like that*.” Some caregivers employed a proactive approach, like pre-placing healthy snacks, saying, “*I’ll put grapes next to her, so when she says she’s hungry, they’re already there*.” (Caregiver 19). To make healthier foods more appealing, caregivers often incorporated vegetables into favorite dishes, like Caregiver 20 noted, “*She won’t eat broccoli on her plate, but if it’s in an egg casserole, she doesn’t even blink an eye.*” Additionally, involving individuals with SMS in meal preparation, such as packing their own lunches, helped some individual increase acceptance of healthier food choices. Implementing healthy options early in life was felt to be important to some families, as Caregiver 9 described, “*She’s never had soda or Skittles, so she doesn’t even want them. She’ll just give them to other people*.” Despite these strategies, challenges emerged when aligning family food habits with the dietary needs of individuals with SMS. Caregiver 14 expressed, “*I wish I could change everyone’s eating habits in my house because we are from Texas, and it’s a meat-and-potatoes culture.*” These experiences reflect the caregiver’s consistent efforts to foster healthier choices while navigating individual preferences and family dynamics.

#### Satiety optimization (12/23 [52.2%] caregivers representing 11/21 [52.4%] individuals with SMS)

Caregivers utilized multiple strategies to curb hunger, including medications and dietary interventions. Dietary adjustments played a crucial role, as Caregiver 9 shared that a low sugar diet “*changed all the cravings dramatically.*” Hydration strategies were also employed to aid satiety, with Caregiver 22 explaining, “*I try to give him three cups of water through his G-tube before a snack so that he feels fuller.*” Additionally, medications were utilized as Caregiver 3 shared, “*The Ritalin was better. She wasn’t hungry at all*.” Some caregivers also explored non-prescription treatments, like apple cider vinegar, probiotics, and fiber. These strategies emphasized the importance of personalized approaches and the blending of medical, dietary, and behavioral strategies to optimize care for individuals with SMS.

#### Intentional portioning and guidance (12/23 [52.2%] caregivers representing 11/21 [52.4%] individuals with SMS)

Caregivers emphasized the role of portion control in fostering healthier eating habits for individuals with SMS. Many took an active role in regulating serving sizes, particularly in situations where overeating was likely to occur. Caregiver 1 explained, “*If there’s a buffet, one of us is going to follow her to make sure she grabs what [we want her] to have*.” Others relied on structural limits, such as restricting second helpings or holding back a portion of the first serving in order to be able to give a “second serving”. Additionally, portioning techniques were used as Caregiver 15 stated, “*she can only take a fist-sized quantity.*” Some caregivers introduced pre-portioned snacks to provide a sense of autonomy while maintaining control, with Caregiver 12 stating, “*[We divide a frozen bag of peas into 5 bags each day.] She knows to ask, and she can get up to five bowls of frozen peas.*” A few reflected on the challenge of introducing structured portioning later in life, noting the benefits of reinforcing healthy eating concepts earlier. These strategies allowed caregivers to create a balance between food regulation and individual independence.

#### Organizing theme # 3: setting the stage for success (16/23 [69.6%] caregivers representing 16/21 [76.2%] individuals with SMS)

Because individuals with SMS faced challenges with self-regulation, caregivers played a crucial role in creating predictable and empowering boundaries to promote successful eating habits. Through anticipatory guidance and conditional compliance strategies, caregivers proactively managed expectations, thus allowing individuals to make choices within preset boundaries. By consistently using positive language and reinforcement, caregivers fostered cooperation and compliance. Across home, school, and community contexts, coordinated caregiver approaches were essential in maintaining consistency across different settings, reinforcing empowerment through stability and shared understanding.

#### Anticipatory guidance and positive language (14/23 [60.9%] caregivers representing 14/21 [66.7%] individuals with SMS)

Encouraging cooperation and reducing food-related conflicts often required a nuanced approach, with caregivers emphasizing proactive positive language and anticipatory strategies to guide behavior. Providing structured choices helped mitigate potential meltdowns, as Caregiver 4 described, “*If I tell her, ‘If you eat your donut now, you’re not going to have it for snack time or dessert,' she’s usually okay with that and moves on.*” In a similar vein, gentle reminders of past consequences were helpful, like “*reminding her that she gets sick [when she eats more than one hot dog]” (Caregiver 20).* Many avoided direct refusals, as Caregiver 23 noted, “*We try not to say the word ‘no’ in my house. It just seems to bring out the deranged lunatic.”* Instead, caregivers used phrasing such as *“‘all done” (Caregiver 23), “Okay, we’ll come back to that later” (Caregiver 5), or “I don’t know yet” (Caregiver 9).*” Furthermore, setting expectations in advance and reinforcing rules helped caregivers maintain structure and avoid disruptions. These approaches transformed everyday interactions into opportunities for guided autonomy, underscoring the significance of intentional communication to promote cooperation.

#### Clear and firm boundaries (5/23 [21.7%] caregivers representing 5/21 [23.8%] individuals with SMS)

Caregivers established clear and firm boundaries to provide the predictability necessary for self-regulation. Many caregivers developed food “rules” to delay or limit food intake while also lessening conflicts. As noted earlier, Caregiver 12 created a rule that allowed her child to eat up to five bowls of frozen peas. Caregiver 10 used the grams of sugar in a food to determine its acceptability and noted, “*She reads the labels. She looks. She’ll say, ‘Hey Mom, I can have three of these. There’s only seven grams of sugar’.*” Additionally, Caregiver 3 developed a rule around spots on bananas to delay her child eating them right after purchase and noted, “*She knows where they are and then, ‘Okay. I see brown spots now. Can I have one?’*” In all of these examples, the black/white nature of the rules allowed for the individual with SMS to maintain a sense of autonomy and develop independence within these known parameters.

#### Coordinated caregiver approach (5/23 [21.7%] caregivers representing 5/21 [23.8%] individuals with SMS)

Although only 5 out of 23 caregivers explicitly contributed to the need for a coordinated caregivers’ approach, their insights highlighted a critical need for consistency across different environments. Caregiver 4 planned to create a personalized guide, explaining, “*Anyone that’s going to be a part of her life outside of my home… you’re going to read this, you’re going to agree to my terms and conditions*.” Others emphasized the necessity of collaborating with authority figures, such as Caregiver 20, who stated, “*We have to talk to her bosses… and not let them allow her to go and just buy $5 worth of soda*.” Furthermore, having reliable support during challenging moments was imperative, as Caregiver 13 noted, “*I always have to have somebody there with me for these meltdowns*.” This coordinated approach supported the individual’s ability to generalize his/her behaviors across different contexts, reflecting the use of empowerment outside of the home environment.

#### Organizing theme # 4: managing challenges and resistance (15/23 [68.2%] caregivers representing 14/21 [66.7%] individuals with SMS)

Management of behaviors is a significant aspect of day-to-day life for caregivers of persons with SMS. Negative behaviors may include self-injury, aggressive and combative actions, and/or temper tantrums at any age and are exacerbated by anxiety, unexpected change in routine, and cognitive abilities [14]. Thus, caregivers employed intentional strategies to guide autonomy in their child to manage these challenges. This subtheme underscores the importance of emotional regulation, redirection, and adaptive responses to mitigate conflicts.

#### Emotional regulation and attention redirection (14/23 [60.9%] caregivers representing 13/21 [61.9%] individuals with SMS)

Caregivers recognized the need for effective strategies to manage emotional reactions and refocus attention during food-related challenges. Redirection served as a mechanism to both calm the individual and to gently provide a path toward empowerment. Caregiver 4 shared, “*We have to redirect her focus and give her options if she’s really pushing it…I try to gently guide her to something I would prefer her to eat.*” The same caregiver noted, “*We can usually get her focus to shift onto something else if she’s consistently asking for food.*” Some caregivers incorporated mindful eating methods to help children slow down and stay present during meals, which helped individuals connect choices with outcomes. Caregiver 11 explained, “*I’ll have him close his eyes and really enjoy his meal, trying to keep him in the moment.*” These strategies demonstrate the value of redirection and mindfulness techniques as strategies to support autonomy and agency while supporting emotional regulation.

#### Navigating bargaining and resistance (6/23 [26.1%] caregivers representing 6/21 [28.6%] individuals with SMS)

Handling bargaining and resistance around food was a large issue as Caregiver 2 described, “*If we go eat or if she has a soda or something that she’s not supposed to have, it becomes such a huge battle to make her not drink what she’s not supposed to have.*” To address this challenge, caregivers used structured consistency, rather than punitive control, to reduce conflict. Many of the above strategies to structure the environment or expectations were used to prevent access altogether (for example locking away food, not buying specific foods, or setting clear guidelines on appropriate food). Alternatively, some caregivers reframed delaying food access as a logical outcome from the individual’s actions by tying food access to completion of a specific task, with Caregiver 3 noting, “*Until your room is clean or’til the meal is put out, you can’t…*.” Some used reflective questioning and their previously discussed “rules” to prompt self-reflection and empower decision-making, as Caregiver 3 shared, “*Ask the questions back. ‘Can I have Mountain Dew?’ ‘I don’t know. Can you have Mountain Dew?’ ‘Yes. Yes.*” These insights illustrate the personalized strategies caregivers used to maintain boundaries in food-related situations while reinforcing empowerment and problem-solving.

## Discussion

**Fig. 2:**
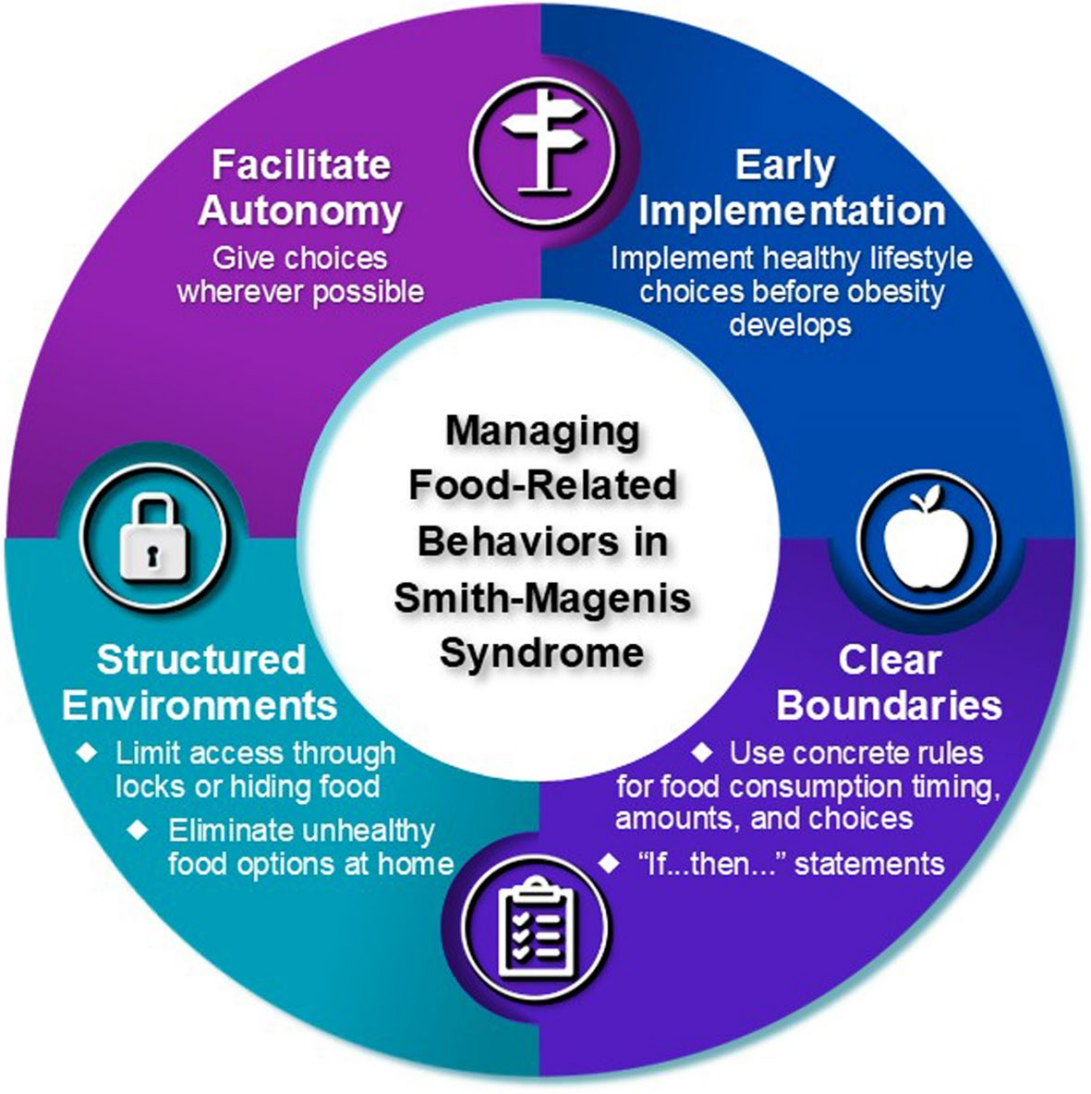
Overall general recommendations for beneficial strategies to aid in reducing disruptive food-related behaviors in individuals with SMS. Key practice points include: (1) early implementation of healthy food options and routines before obesity develops; (2) utilizing clear boundaries with predictable rules as well as “If…then…” statements to allow for choice within approved options; (3) structuring environments at home/work/school to remove or limit access to unapproved food options; (4) giving at least two options for the individual to choose from whenever possible

This study offers a unique insight into the variety and complexity of strategies utilized by caregivers to help their child manage food-related behaviors. Successful strategies often tapped into the unique aspects of the syndrome, such as concrete thinking, to provide boundaries that would be accepted by the individual with SMS, while also offering empowerment and consistency. The most prevalent and effective caregiver recommendations communicated in this study are summarized in Fig. 2 and listed below.

1. Facilitating autonomy by giving healthy choices and supporting inclusion in decision-making can increase independence and reduce conflict over time.
2. Most caregivers utilized some form of structured environment to physically limit access to food, including locks, storing food in hidden locations, and eliminating unhealthy food options.
3. Setting expectations further supports the structured environment by the use of clear and consistent boundaries to indicate what food can be eaten and when food can be eaten.
4. Finally, awareness of the significant risk for obesity in persons with SMS is critical. Thus, early implementation of healthy lifestyle choices and direction, as appropriate for age, can reduce both the risk of obesity and negative and challenging food-related behaviors.

The study’s findings align closely with the Socioecological Model, illustrating how caregiver strategies operate across multiple layers of influence to support balanced eating and behavioral regulation in individuals with Smith-Magenis syndrome (SMS). At the individual level, structured routines, portion control, and reinforcement of healthy choices promote self-regulation and reduce disruptive food-related behaviors. The interpersonal level is reflected in caregivers’ use of consistent boundaries, anticipatory guidance, and positive communication to reduce conflict within the family. At the organizational and community levels, caregiver coordination with their child’s school and work environments promote consistency and reinforce desired food-related behaviors. Together, these findings emphasize that empowering individuals through structured boundaries requires actions across different environments to enhance autonomy and nutritional well-being.

General advice from several caregivers emphasized that strategies must be flexible, as food-related behaviors in individuals with SMS often improve or change over time. Our study gathered information on strategies that had been used at any point in the individual’s life, and we did not explicitly collect information about the individual’s age when any particular strategy was mentioned. However, effective strategies employed by any caregiver must be tailored to the individual’s developmental stage. Thus, it is likely that some of the flexibility necessary for food-related management over time stems from age-related changes in development. Regardless, many caregivers described the necessity of tailoring approaches through trial and error to determine what worked best for their child. Implementing strategies that had realistic expectations and aligned with family routines and meal habits ensured better adherence and cooperation. Moreover, caregivers acknowledged that successful food-related management strategies evolved as individuals with SMS matured, necessitating ongoing adaptation and adjustment.

For many individuals, strategies utilized to encourage positive general behavior had to be modified for food-related behavior. Individuals with SMS struggle when they are out of their typical routine [14]; thus, providing a “heads up” about upcoming changes through proactive statements were commonly effective. However, discussing upcoming food options/plans was not universally beneficial and, in some cases, specifically avoided due to perseveration about the food-related event and disappointment or negative behavior if plans were not as expected. Thus, the way in which caregivers communicated was a crucial strategy. Caregivers avoided directly saying “no” and instead utilized carefully constructed boundaries, as noted above. For example, utilizing a “rule” like five bags of frozen peas or < 7 g of sugar in a snack, can be used to reflect back to an individual when they ask for additional food (i.e. “Can you have that? I don’t know – how many have you had” or “How many grams of sugar does it have?”). Caregivers also became masters of redirection and deflection to avoid battles. The full list of strategies employed by caregivers is presented in Supplemental Table 1.

Similar to PWS, caregivers of individuals with SMS often utilized strategies to lock/hide/limit food availability [7, 10]. Recently, a questionnaire (the Food Safety Zone) was developed for the PWS population to measure food security strategies [6]. This new measure has questions about strategies that are also seen in our population. For instance, the questionnaire asks if caregivers alert other individuals to the need for their child to have limited food, if food is locked away, if money is kept out of the child’s reach, and if the child is specifically excluded from going to the grocery store. However, as we have noted in our previous work, individuals with SMS do not display classic features of hyperphagia but rather have a differential expression focused on food perseverations/obsessions about specific foods [7]. Thus, some families restrict access only to specific/desired foods. Additionally, the Food Safety Zone asks about checking the individual themselves or their belongings for hidden food. These strategies were not discussed in our interviews, although they were not explicitly asked about. Interestingly, some of our caregivers revealed that their children with SMS would proudly tell their parents they got access to prohibited items or leave clear evidence of eating food, suggesting that hiding food may not be as important in the SMS population. Thus, it would be interesting to see how caregivers of individuals with SMS respond to the Food Safety Zone questionnaire compared to its intended population of caregivers of individuals with PWS. Overall, the importance of food and cognitive delays in both SMS and PWS likely lead to similarities in the need for food-related strategies, but the differences in the hyperphagia phenotype, likely driven by differences in underlying physiology, require different strategies between the two groups.

Across different syndromes involving cognitive and behavioral dysregulation, structured caregiver strategies are a common goal to reduce uncertainty. As noted above, in PWS, caregivers lock away food and have strict mealtime routines. Angelman, Williams, and Fragile X syndromes utilize structured routines to mitigate anxiety and emotional dysregulation [5, 12, 17], although these are typically not in the context of food. One distinction with our study is that we noted an explicit intent of caregivers to provide empowerment through structure to foster self-regulation for the individual with SMS. While this may be true in other syndromes, there is no literature explicitly analyzing caregiver strategies through this lens. We would anticipate that strategies which provide empowerment would be beneficial for all but the most profound neurodevelopmental syndromes.

Caregivers who set boundaries, locked away food, or made significant dietary changes early in their child’s life felt that the early interventions were key. This has also been noted in the PWS population [6] and is in line with traditional developmental behavior literature regarding the importance of setting boundaries early in life [4]. In general, all individuals benefit from a healthy lifestyle. Implementing structured strategies, such as portion control, meal planning, and reinforcing healthy eating habits early in life were identified as key factors in achieving long-term success. Caregivers felt early intervention and consistent reinforcement helped individuals with SMS develop better self-regulation skills, ultimately reducing food-related challenges as they grew older. Thus, early discussions by healthcare teams regarding holistic approaches to nutrition may be crucial in individuals with SMS and have the added benefit of preventing or reducing food-related challenges. However, given the highly individualized expressions of food-related behaviors in individuals with SMS, flexible and tailored interventions must be employed to optimize nutrition and decrease conflicts.

Despite caregivers finding success utilizing many strategies, other caregivers found these same approaches ineffective. As noted above, some families found involving their child in meal preparations exacerbated unwanted behaviors. Some caregivers reported that medicines to treat weight or appetite were ineffective or had limited effectiveness for their child. Additionally, the abundance of available food within the household created challenges in restricting or limiting access. These variations in success highlight the need for an individualized approach to effectively mitigate food-related challenges.

Given that hyperphagia stems from disruptions in the normal activity of satiety pathways, it is not surprising that medications that may improve the function of these pathways may be helpful [9]. Surprisingly, few of our subjects mentioned the use of medicines as a helpful strategy. The lack of use of medicines as a strategy is likely multifactorial. However, it is essential to note that there are known biases within the medical community against obesity, which may limit the use of anti-obesity medicines [13]. Our work did not delve deeply into whether families had discussed weight-lowering medicines with their medical teams; thus, we do not know if the lack of medicine use was due to family preference or provider silence. Additionally, in pediatrics, effective weight management medicines have only been available for a few short years and are only approved for 12 years of age and older, and therefore, it is possible that the lack of use of medicines reflects the lack of FDA-approved therapies. Lastly, genetic forms of obesity may respond differently to anti-obesity medications and may require combination therapy and utilizing different benchmarks for success, such as behavior changes or reduced rate of weight gain instead of body mass index (BMI) changes [9]. This study further highlights the clear need for better understanding of both medical and non-medical therapies to address weight gain in SMS population.

Since our study recruited primarily through the SMS patient advocacy group, PRISMS, it is unknown if families involved less in research would utilize the same strategies. Additionally, since our sample was mostly non-Hispanic white individuals, it is unknown if other races and ethnicities would have different methods of mitigating behavior. However, it is likely that all individuals with SMS regardless of race, ethnicity, or socioeconomic status would likely respond positively to strategies that are empowering. We also acknowledge that this study utilized a small sample size, common for rare diseases. Due to this, it is possible that reporting biases in our subjects may not be generalizable to the entire SMS population.

## Conclusion

A structured, comprehensive approach that allows for autonomy while incorporating portioning, environmental controls, coordinated caregiver communication, and holistic nutritional strategies holds promise for mitigating maladaptive food behaviors and can reduce the risk for obesity. However, successful strategies often require orchestrating situations so that individuals feel empowered to act within clearly defined boundaries, while also allowing for flexibility over time. As noted earlier, key recommendations relating to each of our themes are presented in Fig. 2. Given that caregivers felt earlier implementation improved compliance and supported better daily living, future research is needed to determine how to help families define and implement successful personalized strategies for the individual with SMS to support autonomy and healthier living.

## Supplementary Information

Supplementary Material 1.

## Data Availability

All data supporting the findings of this study are available within the paper and its Supplementary Information.
