# Supplementary material for: Empowering through structured boundaries: an integral model for fostering balanced eating and nutritional well-being
Source: J Rare Dis (Berlin). 2026 Jan 13;5(1):2. doi: 10.1007/s44162-025-00145-3 (PMC12799733; doi:10.1007/s44162-025-00145-3)
Supplement: Supplementary file 1 — Supplementary Material 1. [file 44162_2025_145_MOESM1_ESM.docx]

| **Supplemental Table 1: List of Participating Caregivers' Strategies (Advice)**  *Note, these are suggestions from caregivers and should be utilized in conjunction with an individual’s care team and within each family’s comfort zones. Some strategies are opposites of each other, reflecting the need for individualization as well as adapting strategies to an individual’s developmental stage.* | |
| --- | --- |
|  | **General Considerations** |
|  | Start food routines and expectations early, as self-regulation often improves with age. |
|  | Avoid pressuring individuals with SMS to eat if they are not hungry to prevent overeating and promote a healthy food relationship. |
|  | Communicate the plans and the expectations in a transparent fashion before events to reduce anxiety and support behavioral regulation. |
|  | Be aware of the “fairness perception” in many SMS individuals and recognize they feel that food/treats should be equally distributed among all present. |
|  | Avoid labelling food as “good” or “bad” to promote a neutral and balanced food environment. |
|  | Use visual aids, such as clocks, to support when meals/snacks will occur and reduce food-related anxiety. |
|  | Use rewards or reinforcements (such as preferred items or electronics) strategically to encourage cooperation and desired behaviors. |
|  | Avoid announcing meals in advance if it leads to fixation or resistance; instead, provide neutral responses to reduce related anxiety (like “I’m not sure yet”). |
|  | Use repeated, calm reminders (like “Your body's telling you you're hungry. It’s lying. You're not hungry”) to address perceived hunger shortly after meals. |
|  | Strike a balance between independence and gentle boundaries around food access, without resorting to extreme control if appropriate. |
|  | Encourage the use of non-food rewards, such as preferred hobbies or activities, as healthy alternatives to food. |
|  | Use humor (or sometimes lying) to prevent emotional outbursts or calm emotions down. |
|  |  |
|  | Other Medical Considerations |
|  | Be mindful that some medications used for behavior control may cause weight gain. |
|  | Treat yeast overgrowth under a doctor's supervision. |
|  | Use laxatives that can be added to food and beverages to avoid constipation. |
|  |  |
|  | Mealtime Considerations |
|  | Allow the individual with SMS to eat alone (may even be at a different time than the family). |
|  | Allow flexibility with mealtimes and let the SMS individual eat when they express genuine hunger. |
|  | Use nonverbal cues to encourage chewing and swallowing before speaking during meals (like pointing to one’s cheek so the child knows to continue chewing before speaking). |
|  |  |
|  | Meal Preparation |
|  | Minimize food-related discussions and preparation involvement if they cause anxiety or lead to meltdowns. |
|  | Allow participation in food prep with support, focusing on inclusion rather than skill perfection. |
|  | Let them help pack lunch bags. |
|  |  |
|  | Improving Dietary Quality |
|  | Dietary Modifications |
|  | Reduce/eliminate the use of processed food. |
|  | Consider adjusting the diet until you reach the regimen that works for the individual with SMS. |
|  | Avoid foods that contain maltodextrin. |
|  | Implement organic food as much as possible in the daily diet routine. |
|  | Try different diets such as gluten-free, dairy-free, low-carb, and sugar-free. |
|  | Replace regular milk with plant-based milk. |
|  | Adjust the diet based on observed effects, as one parent noted, “the diet seemed to help with a lot of the respiratory issues.” |
|  | Be mindful of the type of food that triggers emotions, especially fast food. |
|  |  |
|  | Implementing Health(ier) Foods |
|  | Offer healthy foods ahead of time so they are readily available when hunger strikes. |
|  | Start the meal with healthy food first. |
|  | Offer healthier versions of regular food, such as burgers without buns, and vinegared cucumber instead of pickles. |
|  | Encourage hydration by providing a bottle of water in the lunch bag or at meals. |
|  | Allow a free range of fruits and vegetables. |
|  | Decrease process foods with “food rules” like “If you can pick it from a tree, pull it from the ground, or kill it, we can eat it”. |
|  | Offer fruit instead of sugary food. |
|  | Introduce more vegetables and fruits at an earlier age |
|  | Implement intentional eating at an early age. |
|  | Explore different cuisines if the local one is missing vegetables and fruits. |
|  | Avoid making or buying sweets, such as cookies, cupcakes, or Pop-Tarts. |
|  | Purchase the food in the exterior aisles of the grocery store for meal prep. |
|  | Incorporate more vegetables, such as broccoli, into other favorites, like casseroles. |
|  | Start avoiding sugary foods such as soda and candies at an early age. |
|  | Replace conventional snacks, such as chips, with healthier options, like sweet potato chips or plant-based protein chips. |
|  | Avoid offering food or drinks with caffeine, to help manage behavior and energy levels. |
|  | Allow controlled access to healthy food, such as fruits and vegetables, and caffeinated beverages. |
|  | Leave an accessible healthy snack (such as a banana) for a midnight raid. |
|  | Create a snack box for snacks that are under 100 calories for daily access. |
|  |  |
|  | **Reducing Food Consumption** |
|  | Restricted Access at Home |
|  | Lock and limit access to refrigerators, pantries, and cabinets using bicycle chains, locks, dial padlocks, safes, or baby gates if appropriate. |
|  | Consider using multiple locks on all doors and cabinets that have access to food. |
|  | Hide treats in unusual spots such as ovens, a safe box, or a pocketbook/purse. |
|  | Put desired/treat food on higher shelves. |
|  | Prevent night raids by locking the individual with SMS in at night, whether by using a safety sleeper, locking the bedroom door, or securing the food area. |
|  | Consider keeping the individual with SMS out of the kitchen if food prep becomes overwhelming. |
|  | Designate a specific food area for the SMS individual, such as a designated shelf, snack box, or designated eating area. |
|  |  |
|  | Reducing Food Consumption Outside of the Home |
|  | Avoid buffet dining/restaurants. |
|  | Avoid bringing the individual with SMS to the grocery store if they exhibit increased control-seeking behavior or emotional outbursts. |
|  | Limit money allowances. |
|  | Avoid showing the individual with SMS the room service/charging the room when at hotels; instead, pay in cash. |
|  | Work with individuals with SMS’s caregivers and teachers to set clear, consistent boundaries and routines around food, such as sitting slightly apart during meal and snack times. |
|  | Create a “101 Guide” for your child detailing food preferences and behavioral needs for anyone involved in their care. |
|  | Communicate with others, like employers, to prevent unsupervised food access. |
|  | Engage caregivers or staff to help monitor portions and prevent overeating in group settings. |
|  |  |
|  | Redirecting and Delay Techniques to Limit Food Consumption |
|  | Shift individuals with SMS attention to food availability timing by directing them to focus on each meal separately. E.g., if the individual with SMS starts the day asking: “When are we going to eat dinner? What's for dinner?” consider answering: "Let’s start with breakfast first”. Another example: if the SMS individual asks for the next meal when he/she just consumed one, consider replying: “Well, we just had breakfast 2 hours ago. Let's just wait until about 2:00.” |
|  | Create explicit rules around food: i.e. bananas can be eaten once they have spots, snacks can only be consumed if they have less than 7 grams of sugar etc. |
|  | Use early redirection strategies with cause-and-effect reasoning to manage fixation and impulsive behavior (i.e. “If you eat that now, then you can’t have dessert later”). |
|  | Redirect calmly and avoid taking items away abruptly to minimize emotional escalation. |
|  | Offer delayed gratification (e.g., "we'll come back to that later") to reduce immediate food-seeking behavior |
|  | Avoid using the word “no”; instead, use alternatives like “not right now” or “all done” to reduce emotional triggers. |
|  | Use bargaining such as: "you can't eat until we're done with this”, “until your room is clean”, or “until the meal is put out." |
|  | Promote self-regulation by using reflective questioning (with previously set guidelines around when food can be consumed), e.g. (child) “Can I have Mountain Dew?” (parent) “I don't know. Can you have Mountain Dew?” |
|  | Provide black and white explanations for questions about why food is being denied. |
|  | Recall past experiences like “Remember how you felt the last time you ate…” |
|  |  |
|  | Portion Control |
|  | Monitor individuals with SMS meal portions at restaurants, especially buffets. |
|  | Pre-portion healthy snacks into a set amount (e.g., two portions) and encourage the individual with SMS to consistently consume the same number daily. |
|  | Teach and encourage the individual with SMS to use hand-based portioning control to measure the meal portion (i.e. “size of your fist”). |
|  | Encourage individuals with SMS to stick to the initial one-portion and avoid offering second servings. |
|  | Divide the meal portion into two smaller servings to create the illusion of seconds without increasing the total amount. |
|  | Introduce the healthy plate model early to support portion control habits. |
|  | Use smaller serving plates. |
|  | Encourage label reading. |
|  | Use natural consequences (e.g., “too much may make you sick”) to encourage moderation. |
|  |  |
|  | Slowing down eating and improving satiety |
|  | Use meditation techniques to encourage the SMS individual to slow down while eating a meal or a treat, such as “Close your eyes. Really enjoy it.” or “Enjoy this meal that you're having right now.” |
|  | Encourage increased food awareness and slow down consumption. |
|  | If the SMS individual has a G-tube, consider giving water before meals or snacks to ensure satiety. |
|  | Consult the individual with SMS’s pediatrician or family doctor to explore options for appetite-suppressing treatments, prebiotics/probiotics, and supplements such as apple cider vinegar or fiber. |
